# 882. An Epidemiological Profile of Pediatric Burns in a Regional Catchment Area

**DOI:** 10.1093/jbcr/irag033.368

**Published:** 2026-04-06

**Authors:** Jakob Weirathmueller, Sandra Bojic, Jakob Weirathmueller, Claudia C Malic

**Affiliations:** University of Ottawa, Ottawa, ON; University of Ottawa, Ottawa, ON; University of Ottawa, Ottawa, ON; University of Ottawa, Ottawa, ON

## Abstract

**Introduction:**

Pediatric burns are a significant public health concern. Regional epidemiological patterns are crucial for targeted prevention. This study characterizes the demographic and clinical features of pediatric burns presenting to a specialized pediatric burn center, representing a large cohort analyzed over 9 years.

**Methods:**

A retrospective review of all pediatric burn cases referred to a specialized pediatric burn center over a 9-year period (Jan 2016-Dec 2024) was conducted. Analysis included demographic data, injury circumstances, burn severity, management, and outcomes. Data were analyzed using descriptive statistics, chi-square tests for categorical variables, and t-tests for continuous variables.

**Results:**

The application of first aid decreased significantly with increasing age (*p*=.0005), from 64.3% in infants to 51.5% in adolescents, representing a modifiable risk factor for outcomes. From 2013 pediatric patients referred, most were male (58.4%) with a mean age of 5.1 years. Burn mechanism varied significantly by age (χ^2^ = 115.3, *p*<.001); scald and contact burns predominated in younger children (mean age 5.1 and 4.0 years, respectively), while flame burns occurred in older children (mean age 7.9 years). The hand was the most commonly injured site (42.8%, n = 861). Most burns were partial-thickness affecting 1-5% TBSA (n = 870), with only 66 patients presenting burns over 10% TBSA. Most patients were managed in ambulatory care.

**Conclusions:**

This study describes the epidemiological profile of pediatric burns at a specialized center. The typical patient is a male toddler with a partial-thickness scald or contact burn to the hand. The declining first aid rates with increasing age represent a critical target for intervention, as proper first aid is associated with improved outcomes.

**Applicability of Research to Practice:**

The significant decrease in first aid application with increasing age suggests a critical need for age-appropriate burn prevention and first aid education campaigns targeting families with school-age children and adolescents. These findings also support targeted prevention programs for high-risk groups (toddlers aged 1-5 years) and inform resource allocation for ambulatory burn care services.

**Funding for the study:**

N/A.

